# Investigating the epidemiology and outbreaks of scabies in Japanese households, residential care facilities, and hospitals using claims data: the Longevity Improvement & Fair Evidence (LIFE) study

**DOI:** 10.1016/j.ijregi.2024.03.008

**Published:** 2024-03-16

**Authors:** Yukihiro Yamaguchi, Fumiko Murata, Megumi Maeda, Haruhisa Fukuda

**Affiliations:** Department of Health Care Administration and Management, Kyushu University Graduate School of Medical Sciences, Fukuoka, Japan

**Keywords:** Scabies, Outbreak, Claims data, Prevalence, Residential care facility

## Abstract

**Objectives:**

This study aimed to characterize the epidemiology of scabies and its outbreaks in Japanese households, residential care facilities (RCFs), and hospitals using claims data.

**Methods:**

This descriptive epidemiological study was conducted using claims data from eight municipalities in Japan. Scabies cases were identified using a combination of recorded diagnoses and administered medications. The study period was from April 2015 to March 2019. Outbreaks were defined as ≥2 cases of scabies occurring within a calendar month at a single household, RCF, or hospital.

**Results:**

We identified 857 scabies cases for analysis. The annual prevalence of scabies ranged from 40 to 67 per 100,000 beneficiaries. The annual attack rate of scabies was found to be highest in RCFs (21 per 1000 RCFs), followed by hospitals (11 per 1000 hospitals) and households (0.25 per 1000 households). The annual outbreak attack rate was also highest in RCFs (4.0 per 1000 RCFs), followed by hospitals (1.6 per 1000 hospitals) and household (0.027 per household). The patterns of outbreaks varied widely among the RCFs.

**Conclusions:**

The study showcases the potential of claims data for detecting infectious disease outbreaks, which could provide valuable insight for the future management and prevention of scabies. Infection control of scabies in RCFs is crucial in aging societies.

## Highlights

• Identified 857 scabies cases, with an annual prevalence of 40-67 per 100,000 people.
• Scabies attack rates: 21 per 1000 residential care facilities, 11 per 1000 hospitals, 0.25 per 1000 households.
• Study highlights the claims data's role in identifying outbreaks, and aiding future scabies management.

## Introduction

Scabies is a contagious ectoparasitic skin disease with worldwide distribution and an estimated annual incidence of 191 million cases [1]. This disease accounted for 0.21% of disability-adjusted life-years from all conditions studied by the Global Burden of Disease Study 2015, with higher burdens in East Asia, Southeast Asia, Oceania, and Latin America [2]. At present, there is a dearth of high-quality data on the burden of this neglected disease [2].

In Japan, scabies outbreaks are not uncommon in hospitals and long-term care facilities [3,4]. However, the Japanese government has not designated scabies as a notifiable disease under the National Epidemiological Surveillance of Infectious Diseases Program. For this reason, the trends in scabies prevalence and outbreaks are not monitored through passive surveillance. Moreover, there are no recent national prevalence estimates based on active surveillance or sampling. The Japanese Dermatological Association's Guidelines for the Diagnosis and Treatment of Scabies do not even include an epidemiology section or any epidemiological data [5].

Scabies outbreaks in healthcare institutions can impose a substantial burden on both the patients and the facilities. The main burden on scabies patients is due to the distressing symptoms that they experience as a result of infestation. The predominant symptom is severe pruritus, which can cause extreme discomfort and reduce the quality of life. Moreover, the disruption of the skin's protective barrier can increase the risk of secondary bacterial infections such as impetigo. This can lead to more serious complications, including sepsis, post-streptococcal glomerulonephritis, acute rheumatic fever, and rheumatic heart disease [6]. The burden of scabies outbreaks on facilities arises from restrictions on clinical practice, financial loss, and increased mental and physical strain on staff. Hospital outbreaks in Japan generally last for 2 months [3], and hospitals must sometimes restrict admissions to control outbreaks, resulting in a loss of revenue. Furthermore, hospitals must also bear the cost of scabies treatments and disinfecting clothing, bedding, furniture, and other environmental items that have been contaminated. In addition, healthcare workers are subjected to increased mental and physical stress from intensified contact precautions over a long period of time [3].

Elucidating the epidemiological situation of scabies is the first step to effectively controlling this disease. However, there is a lack of such data from Japan. This study aimed to characterize the epidemiology of scabies and its outbreaks in Japanese homes, residential care facilities (RCFs), and hospitals using claims data.

## Methods

### Data sources

This study was conducted using data provided by the Longevity Improvement & Fair Evidence (LIFE) Study, which is an ongoing multi-region database project created and managed by Kyushu University (Fukuoka, Japan). [7] In the LIFE Study, participating municipalities regularly provide a variety of datasets (e.g., insurance claims data and residence-related information) for the purpose of research.

We acquired claims data on National Health Insurance (NHI) enrollees, Latter-Stage Elderly Healthcare System (LEHS) enrollees, and Long-Term Care Insurance (LTCI) enrollees from eight municipalities. These three public insurance systems are managed by regional governments. The NHI system provides medical care coverage for residents aged ≤74 years, and its enrollees include self-employed persons, unemployed persons, primary industry workers, and their dependents. In Japan, persons employed in companies are covered by employees’ insurance schemes, which include more beneficiaries than the NHI. However, these individuals were not included in the study data. The LEHS provides medical care coverage for residents aged 65-74 years with specific disabilities and all residents aged ≥75 years. In contrast to the NHI and LEHS, the LTCI system only provides long-term care coverage for residents who are certified by municipal governments as having supportive or long-term care needs. The claims data included the following patient-level information from insurance-covered clinical encounters: age, sex, recorded diagnoses, prescribed medications, clinical setting (outpatient or inpatient care), NHI residence codes (identifying each patient's residence), LTCI registration numbers (identifying each patient's RCF), and NHI/LEHS medical facility codes (identifying the facility that treated each patient).

The main specialty of each medical facility that treated a scabies patient was determined using the list of healthcare institutions provided by the relevant Regional Health and Welfare Bureau. These lists are prepared based on the declaration of each institution. The same unique medical facility codes are used in the claims data and this list, thereby enabling data linkages at the institutional level. RCFs were also analyzed according to the following four service types: nursing care for residents of specified facilities, special nursing homes, geriatric health services facilities, and group homes.

This study was approved by the Kyushu University Institutional Review Board for Clinical Research (Approval No. 2020-200).

### Study design

In this descriptive epidemiological study, a scabies case was defined as an individual who had a recorded diagnosis of scabies and had been administered ≥1 scabies medication. From the claims data, cases of scabies were identified from recorded diagnoses using International Classification of Diseases, 10th Revision code B86. In addition, cases of crusted scabies were identified using Japanese disease code 8838781. Scabies medications include ivermectin, phenothrin, and sulfur, which are covered by Japanese health insurance for the treatment of scabies.

We identified scabies cases from among all insurance beneficiaries with claims data between April 2015 and March 2019 in any of the eight participating municipalities. From this initial population, we excluded (i) patients diagnosed with scabies within the previous year, and (ii) patients prescribed any scabies medication within the previous year. We analyzed scabies according to households, RCFs, and hospitals (inpatients only) from fiscal year (FY) 2016 to FY2018. In Japan, an FY spans from April of 1 year to March of the following year.

The prevalence of scabies was calculated from FY2016 to FY2018 using the number of scabies patients as the numerator and the number of insurance beneficiaries as the denominator. Next, we calculated the attack rates of scabies according to households, RCFs, and hospitals. The attack rate was defined as the ratio of individuals affected by scabies within the population that was initially free of scabies. For the calculation of household attack rates, we used the number of households that experienced a scabies patient as the numerator and the total number of households (total number of unique NHI residence codes recorded in the claims data) as the denominator. As each household was identified using its unique NHI residence code, the calculation of the household attack rate was limited to patients covered by the NHI. For the calculation of RCF attack rates, we used the number of RCFs that experienced a scabies patient as the numerator and the total number of RCFs (total number of unique LTCI registration numbers recorded in the claims data) as the denominator. For the calculation of the hospital attack rate, we used the number of hospitals that experienced a scabies patient as the numerator and the total number of hospitals (total number of unique NHI/LEHS medical facility codes recorded in the claims data) as the denominator. We also tallied the number of scabies patients in each household, RCF, and hospital. Finally, we calculated the 3-year mean attack rate in households, RCFs, and hospitals.

### Outbreaks

A scabies outbreak was defined as ≥2 cases occurring within a calendar month at a single household, RCF, or hospital. [8] The outbreak attack rates in households, RCFs, and hospitals were calculated. The numerator of the household outbreak attack rate was the number of outbreaks within a single household (i.e., those having the same NHI residence code), and the denominator was the total number of households (total number of unique NHI residence codes recorded in the claims data). The numerator of the RCF outbreak attack rate was the number of outbreaks within a single RCF (i.e., those having the same LTCI facility registration number), and the denominator was the total number of RCFs (total number of unique LTCI registration numbers recorded in the claims data). The numerator of the hospital outbreak attack rate was the number of outbreaks within a single hospital (i.e., those having the same NHI or LEHS medical facility code). We also calculated the 3-year mean attack rate of each outbreak in households, RCFs, and hospitals.

Finally, we examined the occurrence of cases with crusted scabies and whether they were included in any outbreaks.

## Results

### Population characteristics

In the eight municipalities covered by this study, the number of insured persons with available medical claims data was 429,913 cases and the number of households was 372,509 cases. In terms of the number of institutions, there were 1965 nursing homes and 3285 hospitals. Table 1 shows the patient characteristics and medications of the study population. We identified a total of 857 scabies cases during the study period. The patients’ mean age was 77.4 years, and 66.5% were female. Among all cases, 26.7% were NHI enrollees and 73.0% were LEHS enrollees. Inpatients accounted for 13.1% of all cases. Dementia was the most prevalent comorbidity (33.5%), followed by cerebrovascular disease (31.4%), congestive heart failure (24.7%), and chronic pulmonary disease (23.7%). Overall, the most common medical facility specialties in which scabies patients were treated were internal medicine (46.1%) and dermatology (43.4%). Among the different settings, the most common specialties were internal medicine (54.8%) and psychiatry (33.3%) for hospital cases, internal medicine (71.9%) and dermatology (25.7%) for RCF cases, and dermatology (61.1%) and internal medicine (31.7%) for household cases. Ivermectin (87.7%) was the most commonly used medication, followed by phenothrin (11.8%). Regarding the concordance between recorded diagnoses and scabies medications, we initially identified 1151 patients with a recorded diagnosis of scabies and/or were administered ≥1 scabies medication. Among these, 857 patients (74.5%) were both diagnosed with scabies and received scabies medication, 233 patients (20.2%) had a recorded diagnosis only, and 61 patients (5.3%) received scabies medication only.

**Table 1:** Patient characteristics and medications.

Variable	All cases (n = 857)	Hospitals (n = 112)	Residential care facilities (n = 245)	Households (n = 500)
Female	570 (66.5)	67 (59.8)	195 (79.6)	308 (61.6)
Age, mean [standard deviation] years	77.4 [17.3]	80.8 [11.3]	85.2 [9.2]	72.9 [19.7]
Insurance type				
Latter-stage elderly healthcare system	626 (73.0)	93 (83.0)	227 (92.7)	306 (61.2)
National Health Insurance	231 (26.7)	19 (17.0)	18 (7.3)	194 (38.8)
Comorbidities				
Dementia	287 (33.5)	53 (47.3)	103 (42.0)	131 (26.2)
Cerebrovascular disease	269 (31.4)	51 (45.5)	93 (38.0)	125 (25.0)
Congestive heart failure	212 (24.7)	45 (40.2)	63 (25.7)	104 (20.8)
Chronic pulmonary disease	205 (23.7)	40 (35.7)	51 (20.8)	114 (22.8)
Mild liver disease	138 (16.1)	24 (21.4)	35 (14.3)	79 (15.8)
Peptic ulcer disease	121 (14.1)	15 (13.4)	39 (15.9)	67 (13.4)
Peripheral vascular disease	100 (11.7)	14 (12.5)	39 (15.9)	47 (9.4)
Any malignancy	83 (9.7)	19 (17.0)	17 (6.9)	47 (9.4)
Renal disease	59 (6.9)	14 (12.5)	13 (5.3)	32 (6.4)
Diabetes with chronic complication	39 (4.6)	3 (2.7)	7 (2.9)	21 (4.2)
Rheumatic disease	31 (3.6)	3 (2.7)	10 (4.1)	18 (3.6)
Diabetes without chronic complication	31 (3.6)	8 (7.1)	9 (3.7)	22 (4.4)
Myocardial infarction	25 (2.9)	4 (3.6)	6 (2.4)	15 (3.0)
Hemiplegia or paraplegia	22 (2.6)	2 (1.8)	4 (1.6)	16 (3.2)
Metastatic solid tumor	11 (1.3)	3 (2.7)	3 (1.2)	5 (1.0)
Moderate or severe liver disease	4 (0.5)	2 (1.8)	0(0.0)	2 (0.4)
Medical facility specialty				
Internal medicine	340 (46.1)	51 (54.8)	151 (71.9)	138 (31.7)
Dermatology	320 (43.4)	0 (0.0)	54 (25.7)	266 (61.1)
Psychiatry	33 (4.5)	31 (33.3)	1 (0.5)	1 (0.2)
Plastic surgery	21 (2.8)	0 (0.0)	3 (1.4)	18 (4.1)
Others	20 (2.3)	11 (11.8)	1 (0.5)	12 (2.4)
Medication				
Sulfur	4 (0.5)	2 (1.8)	1 (0.4)	1 (0.2)
Ivermectin	752 (87.7)	89 (79.5)	218 (89.0)	445 (89.0)
Phenothrin	101 (11.8)	21 (18.8)	26 (10.6)	54 (10.8)

All values are presented as n (%) unless specified otherwise.

### Annual prevalence and attack rate

The annual prevalence of scabies among the insurance beneficiaries was 67 per 100,000 beneficiaries (95% confidence interval [CI]: 60-74) in FY2016, 51 per 100,000 beneficiaries (95% CI: 45-58) in FY2017, and 40 per 100,000 beneficiaries (95% CI: 35-46) in FY2018 (Table 2). Table 3 presents the attack rate of scabies at the household, RCF, and hospital level. Scabies affected 166 households, 95 RCFs, and 69 hospitals. Over 3 years, the attack rate decreased from 0.31 to 0.20 per 1000 residents in households and from 24 to 19 per 1000 residents in RCFs. In contrast, the attack rate in hospitals ranged from 9.1 to 14 per 1000 patients and did not exhibit a decreasing trend. RCFs had the highest attack rates. The number of scabies patients according to RCF service type is shown in Supplementary Table 1. Special nursing homes had the most patients (n = 171, 70.0%), followed by nursing care for residents of specified facilities (n = 54, 22.0%). The number of patients per facility was highest at special nursing homes (5.3), followed by group homes (2.8).

**Table 2:** Prevalence of scabies.

Fiscal year	Number of patients (n = 857)	Number of beneficiaries	Prevalence per 100,000 beneficiaries (95% confidence interval)
2016	370 (43.2)	552,194	67 (60-74)
2017	275 (32.1)	536,750	51 (45-58)
2018	212 (24.7)	529,682	40 (35-46)

**Table 3:** Attack rate of scabies at the household, RCF, and hospital level.

Fiscal year	Households			RCFs			Hospitals		
	Number of households with scabies patients (n = 166)	Number of households (n = 372,509)	Attack rate per 1000 households (95% CI)	Number of RCFs with scabies patients(n = 95)	Number of RCFs (n = 1965)	Attack rate per 1000 RCFs (95% CI)	Number of hospitals with scabies patients (n = 69)	Number of hospitals (n = 3285)	Attack rate per 1000 hospitals (95% CI)
2016	70 (42.2)	224,315	0.31 (0.31-0.31)	35 (36.8)	1460	24 (17-33)	30 (43.5)	2121	14 (13-16)
2017	54 (32.5)	217,289	0.25 (0.25-0.25)	30 (31.6)	1511	20 (13-28)	19 (27.5)	2089	9.1 (9.0-9.2)
2018	42 (25.3)	212,924	0.20 (0.20-0.20)	30 (31.6)	1550	19 (13-28)	20 (29.0)	2101	9.5 (9.4-9.6)

CI, confidence interval; RCF, residential care facility.

The number of scabies patients in each household, RCF, and hospital is shown in Supplementary Table 2. Of the 166 households, 146 (88.0%) had only one scabies patient, while 20 experienced ≥2 patients (two patients: 14 households; three patients: four households; four patients: two households). Of the 82 RCFs, 64 (78.0%) experienced only one scabies patient. Five RCFs (6.0%) had ≥10 patients, and the highest number of scabies patients in a single RCF was 61. Next, 112 scabies patients were identified across 57 hospitals. The number of patients ranged from 1-15 among the hospitals. Of the 57 hospitals, 35 (61.4%) had only one scabies patient.

### Outbreaks

Table 4 shows the outbreak attack rate at the household, RCF, and hospital level. Among the households, the outbreak attack rate decreased from 3.1 to 2.4 per 1000 households during the study period. Among the RCFs, the outbreak attack rate ranged from 2.6 to 5.5 per 1000 facilities. When analyzed according to RCF service type, the outbreak attack rate per 1000 facilities was highest at 13 in special nursing homes, followed by 7.7 in nursing care for residents of specified facilities (Supplementary Table 3). Among the hospitals, the outbreak attack rate decreased from 2.8 to 0.48 per 1000 hospitals during the study period (Table 4).

**Table 4:** Outbreak attack rate of scabies at the household, RCF, and hospital level.

Fiscal year	Households			RCFs			Hospitals		
	Number of outbreaks in households (n = 18)	Number of households (n =372,509)	Outbreak attack rate per 1000 households (95% CI)	Number of outbreaks in RCFs (n = 18)	Number of RCFs (n = 1965)	Outbreak attack rate per 1000 RCFs (95% CI)	Number of outbreaks in hospitals (n = 10)	Number of hospitals (n = 3285)	Outbreak attack rate per 1000 hospitals (95% CI)
2016	7 (38.8)	224,315	0.031 (0.013-0.063)	8 (44.4)	1460	5.5 (5.2-5.7)	6 (60.0)	2121	2.8 (2.6-3.0)
2017	6 (33.3)	217,289	0.028 (0.010-0.059)	6 (33.3)	1511	4.0 (3.7-4.2)	3 (30.0)	2089	1.4 (1.3-1.6)
2018	5 (27.8)	212,924	0.024 (0.0077-0.054)	4 (22.2)	1550	2.6 (2.4-2.8)	1 (10.0)	2101	0.48 (0.39-0.58)

CI, confidence interval; RCF, residential care facility.

Supplementary Table 4 presents the number of patients in each scabies outbreak in households, RCFs, and hospitals. Among the 166 households, 16 (9.6%) experienced outbreaks. In each of these outbreaks, the number of patients ranged from 2-4 (two patients: 12 households; three patients: four households; four patients: two households). Among the 82 RCFs, 23 (28.0%) experienced outbreaks. In each of these outbreaks, the number of patients ranged from 2-25. Among the 57 hospitals, 13 (22.8%) experienced outbreaks (two patients: 10 hospitals; three patients: three hospitals). Table 5 summarizes the mean attack rate and the mean outbreak attack rate of scabies in households, RCFs, and hospitals. The highest outbreak attack rates were observed in RCFs (4.0 per 1000 RCFs, 95% CI: 3.8-4.1), followed by hospitals (1.6 per 1000 hospitals, 95% CI: 1.5-1.7). There were 24 patients with crusted scabies (households: 19 patients; RCFs: three patients; hospitals: two patients). However, none of these cases were included in an outbreak.

**Table 5:** Mean attack rate and mean outbreak attack rate of scabies.

	Mean attack rate per 1000 households, RCFs, or hospitals (95% CI)	Mean outbreak attack rate per 1000 households, RCFs, or hospitals (95% CI)
Households	0.25 (0.22-0.29)	0.027 (0.027-0.028)
Residential care facilities	21 (20-22)	4.0 (3.8-4.1)
Hospitals	11 (10-12)	1.6 (1.5-1.7)

CI, confidence interval; RCF, residential care facility.

**Figure 1:**
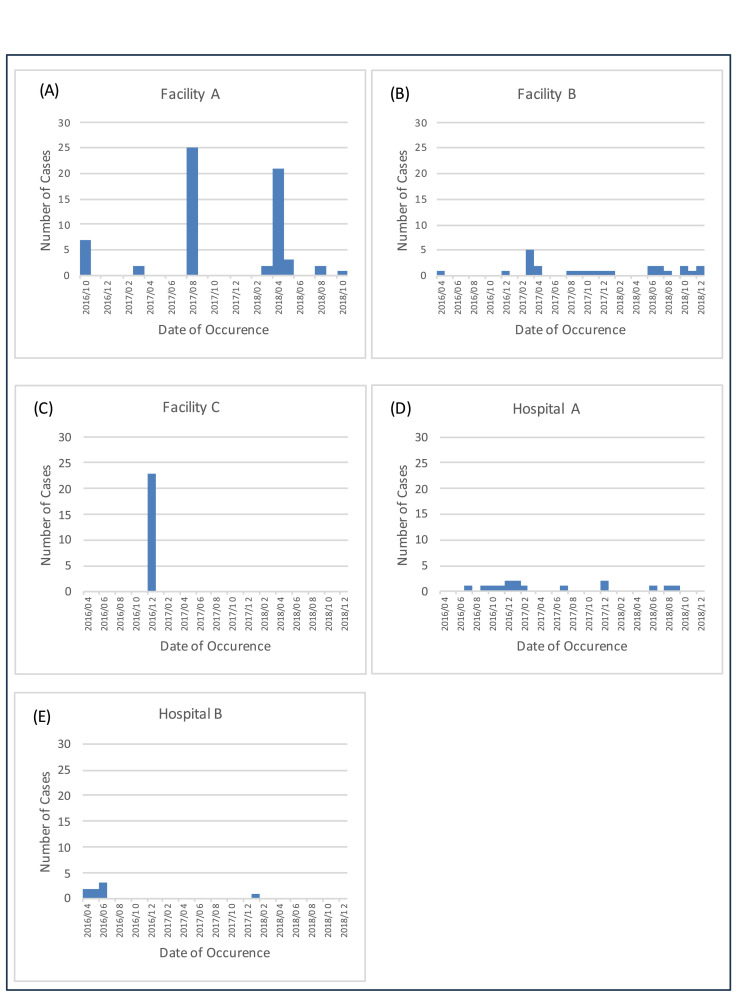
Example of epidemic curves for residential care facilities and hospitals. Sporadic outbreaks occurred in the RCF with the highest number of patients (Facility A). Continuous low-level transmissions occurred in Facility B and Hospital A. The RCF with the third highest number of patients (Facility C) experienced all 23 patients within a single month. In Hospital B seven cases were identified between April and June 2016, but only one case was observed during the rest of the study period (Figure 1e). RCF, residential care facility.

Sporadic outbreaks occurred in the RCF with the highest number of patients (Facility A, Figure 1a). The cases were clustered in August 2017 (n = 25) and April 2018 (n = 23). The RCF with the second-highest number of patients (Facility B) had no surges in scabies cases (Figure 1b). Instead, it experienced continuous low-level transmissions with a total of 25 patients over the study period. The RCF with the third highest number of patients (Facility C) experienced all 23 patients within a single month (Figure 1c). Hospital A, which had the highest number of patients among the hospitals, showed continuous low-level transmissions from September 2016 to February 2017 (Figure 1d). In Hospital B, which had the second-highest number of patients, seven cases were identified between April and June 2016, but only one case was observed during the rest of the study period (Figure 1e).

## Discussion

To our knowledge, this is the first population-based study that estimates the prevalence of scabies in Japan using insurance claims data. In this study, we estimated scabies prevalence and outbreaks over 3 years in patients residing in eight municipalities. The use of claims data enabled the detection of almost all patients.

The annual prevalence of scabies ranged from 0.040-0.067%, which is very low compared to the 0.2-71.4% reported in other countries [9], [10], [11], [12]. However, the use of Japanese claims data to identify scabies cases has yet to be validated, which raises the possibility that there may be inaccuracies in the identified cases. To increase the validity of our scabies case definitions, we used a combination of disease names and medications. The Japanese insurance system covers the use of phenothrin for scabies treatment only and the use of ivermectin for scabies and filariasis; therefore, the influence of indication bias is likely to be small. Furthermore, scabicides are not available over the counter in Japan, and persons with scabies infestations must seek treatment at a healthcare facility. Accordingly, scabies cases are likely to be recorded in insurance claims data. Although several countries have reported increasing numbers of scabies cases, particularly in the last two decades [9], [10], [11],13], our analysis found a downward trend in prevalence over the 3-year study period. Nevertheless, this trend should be confirmed over a longer period.

Approximately half of the inpatients had comorbidities, with dementia being the most common condition. This is consistent with previous reports that dementia is a risk factor for scabies [8,14]. The relatively high prevalence of dementia in our patients may be influenced by the fact that the psychiatric unit was the second most common medical facility specialty in which scabies patients received care. Japan has the highest life expectancy in the world but also has the highest prevalence of dementia [15]. In 2015, the prevalence of dementia in Japanese people aged ≥65 years reached 8% and is expected to increase further [15]. In this context, the prevalence of scabies may also increase in the future.

Numerous scabies outbreaks in healthcare institutions and hospitals have been reported in Japan [3,^4^,14]. However, there is a lack of studies on the prevalence of scabies in such facilities. In our comparison of scabies attack rates in households, RCFs, and hospitals, we showed that RCFs had the highest attack rates by far. This suggests that scabies control in RCFs will become increasingly important in the future, especially with consideration of Japan's aging population.

The patterns of outbreaks among the RCFs were mixed. For Facility A, two outbreaks occurred in 2017 and 2018. In contrast, Facility B experienced multiple small outbreaks over two years without any major outbreaks within a single month; this may indicate a persistent infestation within this facility. Facility C only experienced one major outbreak in a single month with no cases in other months. The epidemic curves of these three RCFs were completely different, indicating that the outbreak patterns vary widely among facilities. Moreover, in sustained low-level transmissions like those in Facility B or Hospital A, there were many months in which only one patient was detected, which does not meet the definition of an outbreak (≥2 cases in a month). To monitor such cases, a different outbreak definition may be necessary, such as counting cases every 3 or 6 months.

Patients with crusted scabies are known to have extremely high infectivity [16]. In a previous questionnaire-based surveillance study of psychiatric and long-term care hospitals in Japan, 32 out of 159 hospitals (20.1%) with scabies patients reported cases of crusted scabies [4]. In our present study, however, crusted scabies cases were not involved in any outbreaks. This may have been due to misdiagnoses or recording errors of crusted scabies. Under Japan's health insurance system, there are no differences in medical fees between scabies and crusted scabies. Consequently, medical institutions lack the incentive to specifically diagnose and record cases of crusted scabies in their insurance claims. There is therefore a need to conduct a validation study of diagnoses in the claims data. In addition, there is also the possibility that scabies patients in the surrounding area were underdiagnosed.

A unique feature of this study is the use of claims data for the detection of infectious disease outbreaks. Although there have been numerous previous reports of scabies outbreaks [3,^4^,14], our search of the literature found no studies that have used claims data to detect outbreaks in households, RCFs, and hospitals. Furthermore, previous reports have generally focused on a small number of hospitals and facilities, whereas our study included all facilities and hospitals within the eight participating municipalities. Therefore, our approach is likely to facilitate the accurate detection of outbreaks. Next, scabies is not usually included in microbiology laboratory databases because it is diagnosed by a physician through examination and microscopy. This makes surveillance difficult without a disease notification system. Therefore, the use of claims data provides another advantage in that it allows outbreaks to be detected despite the lack of a notification system. By circumventing the need to report the occurrence of scabies cases, we can substantially reduce the burden on frontline health workers and insurance administrators. Our findings may contribute to the development of management and preventive strategies for scabies. Future research should be conducted to investigate the characteristics and details of scabies outbreaks. In addition, ongoing monitoring is needed to track the trends in scabies prevalence after 2020 given the potential impact of the COVID-19 pandemic.

This study has the following limitations. Firstly, our analysis was conducted using claims data, which may be vulnerable to misdiagnoses, coding errors, and lack of information on patient compliance. Secondly, the LIFE Study database only included patients who were covered by the NHI, LEHS, and LTCI. Therefore, it did not include individuals who are covered by other types of insurance, such as employer-based insurance. As employer-based insurance provides coverage to working-age persons employed in private companies, its enrollees are generally younger than those enrolled in the three public health insurance systems. Consequently, older persons may be overrepresented in our study population. Thirdly, while the study provides valuable insight into the epidemiology of scabies in Japanese households, RCFs, and hospitals, it does not investigate the factors contributing to scabies outbreaks, such as environmental conditions, staffing levels, hygiene practices, or patient demographics. Understanding these factors is crucial for developing targeted interventions to prevent and control scabies outbreaks. Lastly, the study period covered up to March 2019, which does not include the COVID-19 pandemic period. The pandemic and associated public health measures, such as increased hygiene practices and reduced social contact, could have significantly affected the prevalence and transmission patterns of scabies. Future studies should consider evaluating the impact of the COVID-19 pandemic on scabies outbreaks to provide a more current understanding of the disease's epidemiology.

## Conclusion

The annual prevalence of scabies in households, RCFs, and hospitals in eight Japanese municipalities ranged from 0.040-0.067%. The use of claims data enabled the identification and characterization of multiple outbreaks of scabies. The insights gained from this study may aid the future control of scabies outbreaks.

## Author contribution

YY and HF disigned the study. HF, FM and MM collected the data. YY performed the analysis, and all authors interpreted the results. YY drafted the original manuscript. All authors reviewed and editied the manuscript. The study was surperbised by HF. All authors read the manuscript and approved its submission for publication.

## Declarations of competing interest

The authors have no competing interests to declare.

## Appendix Supplementary materials

Image, application 1

Image, application 2

Image, application 3

Image, application 4
