# Supplementary material for: Investigating the epidemiology and outbreaks of scabies in Japanese households, residential care facilities, and hospitals using claims data: the Longevity Improvement & Fair Evidence (LIFE) study
Source: IJID Reg. 2024 Mar 16;11:100353. doi: 10.1016/j.ijregi.2024.03.008 (PMC11000159; doi:10.1016/j.ijregi.2024.03.008)
Supplement: Supplementary file 1 [file mmc1.docx]

**Supplementary Table 1. Number of scabies patients and attack rate according to RCF service type**

| **Service type** | **Number of**  **patients**  **(n = 245)** | **Number of**  **facilities**  **(n = 82)** | **Number of**  **patients**  **per facility** | **Number of RCFs**  **(n = 1965)** | **Attack rate**  **(95% CI)** |
| --- | --- | --- | --- | --- | --- |
| Nursing care for residents of specified facilities | 54 (22.0) | 39 (47.6) | 1.4 | 781 | 5.0 (3.6-6.7) |
| Special nursing homes | 171 (70.0) | 32 (39.0) | 5.3 | 535 | 6.0 (4.1-8.3) |
| Geriatric health services facilities | 9 (3.7) | 7 (8.5) | 1.3 | 498 | 1.4 (0.57-2.9) |
| Group homes | 11 (4.5) | 4 (4.9) | 2.8 | 151 | 2.7 (0.7-6.6) |

CI, confidence interval; RCF, residential care facility.
