# Supplementary material for: Investigating the epidemiology and outbreaks of scabies in Japanese households, residential care facilities, and hospitals using claims data: the Longevity Improvement & Fair Evidence (LIFE) study
Source: IJID Reg. 2024 Mar 16;11:100353. doi: 10.1016/j.ijregi.2024.03.008 (PMC11000159; doi:10.1016/j.ijregi.2024.03.008)
Supplement: Supplementary file 2 [file mmc2.docx]

**Supplementary Table 2. Number of scabies patients in each household, RCF, and hospital**

| **Number of patients**  **in each household, RCF, or hospital** | **Number of households**  **(n = 166)** | **Number of RCFs**  **(n = 82)** | **Number of hospitals**  **(n = 57)** |
| --- | --- | --- | --- |
| 1 | 146 (88.0) | 64 (78.0) | 35 (61.4) |
| 2 | 14 (8.4) | 4 (4.9) | 13 (22.8) |
| 3 | 4 (2.4) | 4 (4.9) | 3 (5.3) |
| 4 | 2 (1.2) | 3 (3.7) | 4 (7.0) |
| 5 |  | 1 (1.2) |  |
| 8 |  | 1 (1.2) | 1 (1.8) |
| 12 |  | 1 (1.2) |  |
| 15 |  |  | 1 (1.8) |
| 17 |  | 1 (1.2) |  |
| 23 |  | 2 (2.4) |  |
| 61 |  | 1 (1.2) |  |

RCF, residential care facility.
