# Supplementary material for: Investigating the epidemiology and outbreaks of scabies in Japanese households, residential care facilities, and hospitals using claims data: the Longevity Improvement & Fair Evidence (LIFE) study
Source: IJID Reg. 2024 Mar 16;11:100353. doi: 10.1016/j.ijregi.2024.03.008 (PMC11000159; doi:10.1016/j.ijregi.2024.03.008)
Supplement: Supplementary file 3 [file mmc3.docx]

**Supplementary Table 3. Scabies outbreaks according to RCF service type**

| **Service type** | **Number of outbreaks in RCFs** | **Number of RCFs** | **Outbreak attack rate per 1,000 facilities (95% CI)** |
| --- | --- | --- | --- |
| Nursing care for residents of specified facilities | 6 | 781 | 7.7 (7.4-8.0) |
| Special nursing homes | 7 | 535 | 13 (10-16) |
| Geriatric health services facilities | 0 | 498 | 0.0 (0.0-0.0) |
| Group homes | 1 | 151 | 6.6 (5.8-7.4) |

CI, confidence interval; RCF, residential care facility.
