# Supplementary material for: Investigating the epidemiology and outbreaks of scabies in Japanese households, residential care facilities, and hospitals using claims data: the Longevity Improvement & Fair Evidence (LIFE) study
Source: IJID Reg. 2024 Mar 16;11:100353. doi: 10.1016/j.ijregi.2024.03.008 (PMC11000159; doi:10.1016/j.ijregi.2024.03.008)
Supplement: Supplementary file 4 [file mmc4.docx]

**Supplementary Table 4. Number of patients in each scabies outbreak** **in households, RCFs, and hospitals**

| **Number of patients in each outbreak** | **Number of households**  **(n = 16)** | **Number of RCFs**  **(n = 23)** | **Number of hospitals**  **(n = 13)** |
| --- | --- | --- | --- |
| 2 | 12 (75.0) | 9 (39.1) | 10 (76.9) |
| 3 | 4 (25.0) | 6 (26.1) | 3 (23.1) |
| 4 | 2 (12.5) | 2 (8.7) |  |
| 6 |  | 1 (4.3) |  |
| 7 |  | 2 (8.7) |  |
| 19 |  | 1 (4.3) |  |
| 23 |  | 1 (4.3) |  |
| 25 |  | 1 (4.3) |  |

RCF, residential care facility.
